# Department of Therapeutics

**Published:** 1893-05

**Authors:** David Cerna

**Affiliations:** Galveston; Demonstrator of Physiology in the Medical Department of the University of Texas, etc.


### Abstracts of Current Medical Literature.

### DEPARTMENT OF THERAPEUTICS.

Under the charge of David Cerna, M. D. Ph. D., Demonstrator of Physiology in the Medical Department of the University of Texas, etc.

CHLOROFORM FOR MAGGOTS IN THE NOSE.—James P. Kimball, of Fort Clark, Texas (*New York Medical Journal*, March 11, 1893), reports an interesting case of maggots in the nose, in which the action of chloroform in causing the expulsion of the worms was very decided. Other remedies had been employed previously without avail. The pure chloroform or a mixture of it with water, equal parts, was injected into the nostrils, a measure that was followed by the expulsion of the maggots. From a study of the case described, and the brilliant results obtained with chloroform injections, the author concludes that in this remedy "we have a reliable remedy for a disease which, under any other method of treatment hiterto recommended, has usually proved fatal." The author condemns remedial measures recommended by various high authorities, consisting in "inhalations of alcohol, either, turpentine and chloroform; syringing with carbolized solutions or solutions of corrosive sublimate, or decoctions of bitter herbs or tobacco; injections of turpentine or of oil; insufflation of calomel, and pencilings with balsam of Peru;" affirming that such measures "are scarcely any more avail than Mrs. Partington with her broom against the waves of the Atlantic ocean." This may all be well enough in the opinion of Kimball, but the editor of this department has seen excellent results following the simple *insufflations of calomel* in just such cases as that described by Kimball. He also differs with the author quoted in that "it appears to be satisfactorily established that this fly (*Sarcophaga georgina*) deposits its larvæ only on the unsound mucous membrane." I have seen cases in which the larvæ had undoubtedly been deposited in previously healthy mucous membranes. It remains, therefore, to be definitely established whether the fly has a special love for diseased membranes. The cases referred to by Kimball, besides the one reported in detail, do not seem to be sufficiently numerous to warrant him to make a conclusive statement to the effect that the larvæ are deposited *only* on the unsound mucous membrane.

---

ALANGINE.—Alangine is the name applied to an alkoloid found in the root and bark of *Alangium Lamatckii*, Thewaites, a tree belonging to the N. O. cornaceæ. According to Mohideen Sheriff (*Pharm. Ztg.*, p. 17, 1893, *Notes on New Remedies*, February, 1893), it is a safe emetic in doses of 3.0 grammes; in small doses it has an antipyretic effect. It is said to be a good substitute for ipecac, except in cases of dysentery. Alangine, which is regarded as the active principle of the bark, is very bitter and uncrystallizable. According to Schuchardt, it is soluble in alcohol, ether, chloroform and acetic ether, but entirely insoluble in water. It is precipitated from its solutions by mineral as well as acetic, tartaric and oxalic acids, which yield with it crystallizable salts.

---

SALICYLATES IN THE TREATMENT OF PLEURISY WITH EFFUSION.—In an interesting paper, Dr. George Dock, of Ann Arbor, Michigan (*Therapeutic Gazette*, February 15, 1893), reports two cases of pleurisy with effusion, in which the use of the salicylates gave satisfactory results. The author, from personal experience and from a study of the literature of the subject, draws the following conclusions: 1. Salicilylic acid and its salts are among the most effectual agents in the treatment of pleurisy with effusion. 2. In effective doses the remedy is harmless, and with proper selection of the preparation, and care in administration, causes little or no discomfort to the patient. 3. Salicylates act most promptly in pleurisies with serous effusion of recent origin or of long standing, but they are efficient in simple dry pleurisy, and often act favorably in secondary pleurisy. 4. There is no evidence that they are useful in suppurative cases. 5. The drug acts as a diuretic, but may have an effect on the pathological process, or on the cause of the disease. 6. Salicylates have a more marked action in pleurisy than have the diuretics commonly so called. 7. "The duration of the treatment with salicylic preparations is less than with diuretics, common salt or roborant medication."—(Eugster). 8. The remedy can be used at the earliest period, and favorably affects all symptoms. 9. The drug may be given in the form of the acid, or any of its salts, in doses of a drachm of the former, or 1 to 2 drachms of a salt daily. In ordinary cases it is not necessary to give the larger doses, and 60 to 90 grains of sodium salicylate or salol daily may be considered full beginning doses, to be diminished one-third or one-half after the effect is manifest. 10. The ordinary precautions must be observed in giving the drugs, and during their administration the total amount of urine should be measured daily.

---

ICHTHYOL IN GYNECOLOGY.—A careful study of the uses of ichthyol in gynecology warrants Homer C. Bloom, of Philadelphia (*Merck's Bulletin*, March, 1893), to draw the following conclusions: 1. It acts as an anodyne in nearly all pelvic troubles. 2. It is of no value for the cure of chronic endometritis. 3. It is particularly useless in old chronic exudations around the uterus; but in recent pelvic exudations it is an important ally. 4. It is a good anodyne placebo in salpingitis and ovaritis—clearing the way for other appropriate treatment. 5. It deserves to be tried in cancer of the uterus. 6. In the early stages of specific vaginitis, it is second to no other drug.

---

TIN-POISONING.—W. A. Campbell, of Colorado Springs, Col. (*Therapeutic Gazette*, March 15, 1883), reports six cases of tin-poisoning, and summarizing his observations, states: 1. Stannous salts are poisonous to the human system, being similar in their action to the other mineral poisons,—lead, zinc, arsenic, animony, etc. 2. The salts of tin are anthelmintic as well as the powder product. 3. Toxic doses of salts produce symptoms similar to those from ptomaines. 4. Canned-food products may contain stannous salts in poisonous quantities. 5. The danger from this source is increased from exposure to the air; hence all fruits should be emptied from tin cans as soon as opened.

---

THE TREATMENT OF GONORRHEA.—Writing upon the above subject, H. M. Christian, of Philadelphia, (*Therapeutic Gazette*, March 15, 1893,) says that the use of injections prior to the subsiding stage of acute gonorrhea acts, in quite a large proportion of the cases, as an exciting cause in the production of posterior urethritis and epididymitis, and on this account is not to be considered as the best treatment of the disease. All patients at the venereal dispensary of the University hospital are now put upon the internal use of a capsule containing five drops of sandalwood oil and five drops of the oil of copaiba, with one drop of the oil of cinnamon. From four to eight of these are taken for the first three weeks. When the "morning drop" persists, an injection of sulpho-carbolate of zinc and hydrastis is used. It is not claimed for this plan of treatment that it in any way cuts short the duration of the disease, but only that it aids in preventing the frequent occurrence of posterior urethritis and epididymitis, the two most troublesome complications of gonorrhea.

---

THE THERAPEUTIC USES OF MYRRHOLIN.—In the *Centralblatt fur Klinische Medicin*, February 25, there is an abstract of an article by Dr. M. Kohn, published in the *Munehener Medicinische Wochenschrift*, in which the author reports good results in the treatment of eczema narium with an ointment of myrrh, also in that of both simple and fœtid atropic rhinitis with tampons imbued with the ointment. The use of myrrh as a corrigent in the creasote treatment of pulmonary phthisis is said to have proved satisfactory. The preparation employed was myrrholin (a mixture of one part of myrrh and two parts of oil). Capsules, each containing three-tenths of a gramme of creasote and two tenths of a gramme of myrrh were very well borne by consumptives.—*N, Y. Medical Journal*, March 18, 1893.

